# Effect of requiring advanced respiratory support on disaster-related anxiety among caregivers of children with medical complexity: a cross-sectional study

**DOI:** 10.1186/s12889-026-27601-z

**Published:** 2026-05-07

**Authors:** Hisao Nakai, Shiro Ozasa, Yukari Matsumoto, Keiko Takushima

**Affiliations:** 1 Faculty of Nursing, University of Kochi, 2751-1 Ike, Kochi, 781-8515 Japan; 2 Center for Children’s Medical Homecare, Kumamoto University Hospital, 1-1-1 Honjo, Chuo-ku, Kumamoto, 860-8556 Japan; 3 School of Nursing, Faculty of Medicine, Fukuoka University, 8-19-1 Nanakuma, Jonan-ku, Fukuoka, 814-0180 Japan

**Keywords:** Child with medical complexity, Disaster, Information asymmetry, Technology dependence, Caregiver burden, Advanced respiratory support

## Abstract

**Background:**

The population of children with medical complexity (CMC) is increasing globally. Caregivers must provide highly individualized care, and there may be “information asymmetry” between families and support providers. Infrastructure disruptions during disasters, such as power outages and communication failures, threaten the survival of CMC. This study aimed to estimate the independent effect (after adjusting for daily confounding factors) of requiring advanced respiratory support (ARS) on three types of disaster-related anxiety.

**Methods:**

A cross-sectional online survey was conducted from August to October 2025, targeting caregivers of CMC via a nationwide support network in Japan. Participants were categorized into an ARS group (requiring invasive/non-invasive ventilation, high-flow nasal cannula, or frequent suctioning) and a non-ARS group. Anxiety was assessed across three domains: (1) accurate transmission of information to medical professionals met for the first time, (2) transmission of information in situations where communication methods are unavailable, and (3) continuity of care during power outages. With ARS as the primary independent variable, binomial logistic regression was performed to calculate adjusted odds ratios (AOR), controlling for covariates, including caregiver and child ages, evacuation method difficulty, and daily information-sharing satisfaction.

**Results:**

Data from 279 caregivers were analyzed (child mean age: 9.5 ± 7.0 years). Binomial logistic regression showed that requiring ARS had a significant independent effect on anxiety about the accurate transmission of information to medical professionals met for the first time (AOR: 2.60; 95% confidence interval [CI]: 1.13–5.99; *p* = 0.025) and continuity of care during power outages (AOR: 5.87; 95% CI: 2.99–11.52; *p* < 0.001), but not for situations where communication methods were unavailable.

**Conclusions:**

Caregivers of CMC requiring ARS experience significantly greater anxiety during disasters, caregivers of CMC requiring ARS experience significantly greater anxiety about communicating information to medical professionals and continuity of care. To ensure CMC safety, transitioning to digital personal health records to complement oral and paper-based methods should be considered. Bridging these information gaps may reduce caregiver anxiety and facilitate rapid triage during emergencies.

**Supplementary Information:**

The online version contains supplementary material available at 10.1186/s12889-026-27601-z.

## Introduction

In recent years, the number of children with medical complexity (CMC) has been increasing globally, making the management of their complex care an urgent challenge [1, 2]. CMC comprise a highly vulnerable population characterized not only by chronic illness but also by a dependence on multiple medical interventions and life-sustaining devices, necessitating specialized and continuous support [3]. The type of care that CMC require varies widely and is highly individualized, demanding essential support through multidisciplinary collaboration even during stable periods [4, 5]. Consequently, caregivers must manage and share with support providers a substantial amount of information, which tends to become highly fragmented [6, 7].

The vulnerabilities of CMC are greater during intensified disasters. Globally, climate-related disasters are increasing [8]. In Japan, there is frequent extensive damage from major earthquakes, typhoons, torrential rain caused by linear precipitation zones, and record-breaking heavy snowfall [9, 10]. For CMC, these disasters are not merely inconvenient but are substantial threats to their survival and daily lives [11, 12]. Previous disasters, such as Hurricane Katrina, have created life-threatening situations in which power outages and equipment failures necessitated manual ventilation [13]. Similarly, since the Great Hanshin-Awaji Earthquake in 1995, CMC in Japan have experienced critical situations during every major earthquake and disaster [14, 15].

During disasters, major changes to the home care environment can occur owing to infrastructure loss, such as power and communication outages and damage to buildings. The lack of standardized communication processes has been identified as a factor that prevents families from accessing appropriate support during such periods [15]. A previous study found that only 10% of parents of children with disabilities reported having essential contact information in a physical format, such as paper, during a disaster [16]. Furthermore, even during emergency department visits, the inability to effectively communicate concerns or provide oral input about the child (or the failure of such input to be acknowledged) negatively affects subsequent care [17]. These information continuity challenges have a major effect on the maintenance of necessary medical care for CMC during disasters; consequently, they may not only lead to the exacerbation of pre-existing conditions but also threaten the child’s survival.

To protect CMC living in the community, rapid evacuation to a safe location and continuity of appropriate care at the evacuation site are essential, and both factors presuppose the continuity of information about the child. Specifically, this includes information on medical equipment and procedures necessary for survival, specialized human resources, and the physical environment required to support the child [18]. Previous studies have pointed out the challenges of securing medical supplies during disasters and the anxieties and concerns of caregivers of CMC. These include whether the child can be admitted to a hospital if they experience a sudden change in their condition, whether daily routines can be safely maintained at an evacuation center [19], and whether appropriate support from professionals can be obtained [20]. In particular, it has been reported that among caregivers of CMC, anxiety about the continuity of life-sustaining care exacerbates psychological distress [21]. Qualitative research on the relationship between the severity of the condition of CMC and caregiver anxiety has shown that higher care severity necessitates more diverse information, resulting in fragmented information even during normal times and a greater burden on caregivers regarding care coordination [22]. Furthermore, caregivers of CMC with more severe conditions experience a greater burden of having to explain the child’s complete medical history to supporters [23]. To date, no quantitative studies have demonstrated how the extent of medical care needed is specifically linked to various information-related anxieties, such as those concerning accurate transmission of information to medical professionals met for the first time, communication, and power outages. Among CMC, those requiring advanced respiratory support (ARS) are particularly vulnerable during disasters because of their complete dependence on electricity and specialized medical equipment [11, 24]. In existing disaster management policies, the need for ARS is a critical indicator for prioritizing support [25, 26]. Despite this, the specific effect of ARS dependency on caregivers’ disaster-related anxieties, particularly regarding accurate transmission of information to medical professionals met for the first time and care continuity, remains unclear.

The purpose of this study was to estimate the independent effect of requiring ARS on three types of disaster-related anxiety—accurate transmission of information to medical professionals met for the first time, transmission of information in situations where communication methods are unavailable, and continuity of care during power outages—after adjusting for daily confounding factors. The findings could contribute to the development of specific support measures aimed at improving the lack of information continuity and reducing caregiver burden in existing disaster preparedness strategies for CMC. Ultimately, these findings may inform the promotion of comprehensive disaster countermeasures to protect the daily lives of the most vulnerable CMC.

## Materials and methods

### Data collection

The study participants were family caregivers of CMC who agreed to participate following a recruitment call. Recruitment was conducted through the Japan Medical Care Line, a nationwide network in Japan comprising CMC, their families, and supporters, and Famicare, an information platform supporting children with diseases or disabilities and their families. Data collection was facilitated by Japan Medical Care Line and Famicare, both of which invited their members and users to participate via mailing lists and social media.

Survey items were developed based on a comprehensive literature review of previous studies [15, 16, 27, 28, 29, 30]. Rather than using existing scales, original survey items were created to exploratively capture the real-world challenges of information sharing experienced by family caregivers of CMC in Japan. Due to resource and time constraints during the rapid assessment of these urgent needs, a formal Delphi method was not employed. Specifically, the following process was used to ensure content validity and mitigate researcher expectancy effects: (1) a comprehensive literature review; (2) selection of candidate survey items; (3) multiple rounds of discussions among researchers with diverse clinical and research backgrounds to ensure item relevance, clarity, and alignment with the research objectives; and (4) refinement of the content following a pilot survey with 15 collaborators, including colleagues of the researchers and their co-investigators. This collaborative review process, involving stakeholders from different perspectives, was designed to minimize interpersonal influence during item selection. The survey was conducted from August 4 to October 31, 2025. The full English version of the survey questionnaire is provided in Additional File 1.

### Survey items

#### Participant demographics

The characteristics of sex and age were recorded for both caregivers and children.

#### Medical care received by children in daily life

### Number of medical care procedures

The total number of different types of medical care procedures the child routinely received was recorded.

#### Details of medical care

A checklist was used to record the presence of the following types of medical care: mechanical ventilation (via tracheostomy), non-invasive ventilation (e.g., via oral or nasal mask), high-flow nasal cannula oxygen therapy, mechanical insufflation-exsufflation (e.g., Cough Assist, intrapulmonary percussive ventilation), suctioning (oral, nasal, or tracheal), home oxygen therapy, tracheostomy management, enteral nutrition (via gastrostomy, jejunostomy, or nasogastric tube), total parenteral nutrition (via central venous catheter), management of urinary catheterization (clean intermittent or continuous), stoma management (e.g., colostomy or ileostomy), insulin injections, routine blood glucose monitoring, other self-injections, and peritoneal dialysis.

### Methods of evacuating with the child

Participants were asked to select all applicable methods for evacuating with their child during a disaster from the following six options: (1) being carried by a caregiver, (2) manual wheelchair, (3) electric wheelchair, (4) adaptive stroller, (5) walker, and (6) ambulation without assistance.

### Satisfaction with information sharing with support providers

Participants were asked to rate their overall satisfaction with daily information sharing with support providers. Responses were measured on a 5-point Likert scale: “Very dissatisfied,” “Somewhat dissatisfied,” “Neither satisfied nor dissatisfied,” “Somewhat satisfied,” and “Very satisfied.”

Anxiety about information transfer to unfamiliar professionals and continuity of medical care during power outages.

Participants’ anxieties about these factors were assessed using the following three items, each measured on a 4-point Likert scale (“Not at all,” “Not much,” “Somewhat,” and “Significantly”):

Anxiety about the accurate transmission of information to medical professionals met for the first time

Are you anxious about accurately communicating your child’s condition and care requirements to medical professionals meeting your child for the first time (e.g., emergency medical technicians, emergency department physicians, or nurses) during an emergency, such as during an ambulance transport?

Anxiety about the transmission of information in situations where communication methods are unavailable

Are you anxious about reporting your child’s safety or requesting rescue (to family members, supporters, or local government) during a disaster when communication methods (telephone, internet) are unavailable?

Anxiety about continuity of care during power outages

Are you anxious about being able to continue to receive medical care during a power outage caused by a disaster?

### Analysis methods

Continuous variables (caregiver and child age) were summarized as means and standard deviations, whereas categorical variables (e.g., sex) were expressed as frequencies and percentages. Multiple-response items about daily medical care were converted into binary variables. To evaluate the intensity of care, a medical care count was used, defined as the total number of medical procedures each child had received.

### Variable definitions

#### Dependent variables

The dependent variables were the three types of disaster-related anxiety, measured on a 4-point Likert scale. For analysis, responses on these items were dichotomized into “Yes” (Somewhat/Significantly) and “No” (Not at all/Not much).

1. Anxiety about the accurate transmission of information to medical professionals met for the first time (hereafter, “information transfer to unfamiliar professionals”);
2. Anxiety about the transmission of information in situations where communication methods are unavailable (hereafter, “loss of communication methods”);
3. Anxiety about continuity of care during power outages.

The independent variables and covariates were defined as follows:

ARS group: Participants were classified into the ARS group (coded as “Yes”) if the child required at least one high-risk procedure, the interruption of which would directly affect prognosis (e.g., invasive/non-invasive mechanical ventilation, high-flow nasal cannula, mechanical insufflation-exsufflation, suctioning, or home oxygen therapy). All other participants were classified into the non-ARS group (“No”). The rationale for this dichotomous classification was that substantial dependency on electricity and continuous respiratory support is a critical triage and policy threshold in disaster management for CMC (e.g., prioritization for individual evacuation plans and emergency power allocation) [26]. Although caregivers of children in the ARS group inherently experience higher baseline anxiety related to providing daily life-sustaining care, identifying the specific vulnerabilities of this highly power-dependent population is essential for developing targeted and practical disaster countermeasures.

### Evacuation method difficulty

Participants’ evacuation method difficulty was dichotomized according to the presence of physical barriers during disasters (e.g., steps, debris). The “Difficult” group included those using manual/electric wheelchairs, adaptive strollers, or walkers. The “Easy” group included those relying on unassisted ambulation or being carried by a caregiver.

### Information-sharing satisfaction

Responses to this variable were categorized as “Satisfied” (Somewhat/Very satisfied) and “Others” (Neither satisfied nor dissatisfied/Dissatisfied).

### Statistical procedures

To examine the relationships between the binary variables and being in the ARS group, t-tests were used for continuous variables and chi-square or Fisher’s exact tests were used for categorical variables.

Subsequently, binomial logistic regression analysis was performed to estimate the independent effect of requiring ARS on the three types of disaster-related anxiety. Based on clinical significance and previous literature on the vulnerability of CMC [31, 32, 33, 34], ARS group status was defined as the primary independent variable. Evacuation method difficulty, daily information-sharing satisfaction, and caregiver and child ages were included as forced-entry covariates to control for potential confounding. Furthermore, to contextualize the challenges of information transfer, caregivers’ specific concerns and the preparations they engage in to address these (Additional File 1, Q22 and Q23) were descriptively analyzed using frequencies and percentages. Before the analysis, multicollinearity was assessed using the variance inflation factor, which confirmed that all values were < 5. Listwise deletion was used for missing data; therefore, the sample size (n) could vary between the bivariate analyses but remained consistent within the logistic regression. All analyses were conducted using IBM SPSS Statistics version 29 (IBM Corp, Armonk, NY, USA), with a significance level of *p* < 0.05.

## Ethical considerations

This study was conducted in accordance with the Declaration of Helsinki (as revised in Fortaleza, 2013) and was approved by the Ethics Committee of the University of Kochi (Approval No. 250004). All participants were provided with an explanatory document explaining the study’s objectives, the voluntary nature of participation, the guarantee of anonymity, and the protection of personal privacy. This survey was conducted as a completely anonymous study; no personally identifiable information, such as names or contact details, was collected.

Regarding the informed consent procedure, the study purpose and privacy protocols were presented at the top of the online form. Participants were required to check a consent box before proceeding to the questionnaire. Submission of the completed survey was considered as providing informed consent for participation. Participants maintained the right to withdraw and discontinue their response at any time prior to clicking the “Submit” button. However, because all responses were completely anonymous, individual responses could not be identified once submitted. Consequently, participants were explicitly informed in the explanatory document that data withdrawal after submission was not possible. The aforementioned informed consent procedures and the limitations regarding data withdrawal were reviewed and formally approved by the institutional ethics committee.

Clinical trial number: not applicable.

## Results

### Participant demographics and clinical characteristics

The characteristics of the caregivers and their children are summarized in Table 1. The mean age (± standard deviation) of the caregivers was 42.5 ± 8.2 years. Most caregivers were women (*n* = 262, 95.3%); men accounted for 4.7% (*n* = 13). The mean age of the children was 9.5 ± 7.0 years, with an almost equal distribution of girls (*n* = 133, 50.2%) and boys (*n* = 132, 49.8%).

**Table 1:** Demographic characteristics and status of medical care procedures: comparison between the ARS and non-ARS groups

Items	Category		Total			ARS group														
						No (*n* = 69)						Yes (*n* = 210)					*p*-value			
			*n*	%		*n*			%			*n*	%							
Participant demographics																				
Caregiver’s age (mean ± SD)			42.5 ± 8.2			41.4 ± 8.3						42.8 ± 8.1					0.258^a^			
Child’s age (mean ± SD)			9.5 ± 7.0			8.9 ± 6.7						9.6 ± 7.0					0.435^a^			
Caregiver’s sex	Male		13	4.7		5			38.5			8	61.5				0.320^b^			
	Female		262	95.3		63			24.0			199	76.0							
Child’s sex	Male		132	49.8		27			20.5			105	79.5				0.095^c^			
	Female		133	50.2		39			29.3			94	70.7							
Medical care received by children in daily life																				
Number of medical care procedures (mean ± SD)			3.1 ± 1.9			0.8 ± 0.6						3.8 ± 1.6					< 0.001^a^			
Details of medical care																				
Mechanical ventilation (via tracheostomy)			88	31.5		0			0.0			88	100.0				NA			
Non-invasive ventilation (e.g., via oral or nasal mask)			22	7.9		0			0.0			22	100.0				NA			
High-flow nasal cannula oxygen therapy			11	3.9		0			0.0			11	100.0				NA			
Mechanical insufflation-exsufflation (e.g., Cough Assist, intrapulmonary percussive ventilation)			52	18.6		0			0.0			52	100.0				NA			
Suctioning (oral, nasal, or tracheal)			192	68.8		0			0.0			192	100.0				NA			
Home oxygen therapy			108	38.7		0			0.0			108	100.0				NA			
Tracheostomy management			117	41.9		3			2.6			114	97.4				< 0.001^c^			
Enteral nutrition (via gastrostomy, jejunostomy, or nasogastric tube)			218	78.1		41			18.8			177	81.2				< 0.001^c^			
Total parenteral nutrition (via central venous catheter)			6	2.2		2			33.3			4	66.7				0.639^b^			
Management of urinary catheterization (clean intermittent or continuous)			23	8.2		3			13.0			20	87.0				0.150^b^			
Stoma management (e.g., colostomy or ileostomy)			4	1.4		1			25.0			3	75.0				1.000^b^			
Insulin injections			4	1.4		2			50.0			2	50.0				0.256^b^			
Routine blood glucose monitoring			2	0.7		1			50.0			1	50.0				0.434^b^			
Other self-injections			4	1.4		2			50.0			2	50.0				0.256^b^			
Peritoneal dialysis			0	0.0													NA			
Evacuation method																				
Mobility difficulty	Easy		71		25.4	17				23.9		54			76.1			0.859^c^		
	Ambulation without assistance		17		6.1															
	Carrying by a caregiver		195		69.9															
	Difficult		208		74.6	52				25.0		156			75.0					
	Manual wheelchair		71		25.4															
	Electric wheelchair		4		1.4															
	Adaptive stroller		153		54.8															
	Walker		0			0														
Satisfaction with information sharing with support providers																				
Satisfaction with information sharing with support providers	Others (“Very dissatisfied,” “Somewhat dissatisfied,” “Neither satisfied nor dissatisfied,”)		145	52.0			35	24.1				110		75.9			0.811^c^			
	Yes		134	48.0			34	25.4				100		74.6						

^a^Student’s t-test, ^b^Fisher’s exact test, ^c^Chi-square test

*ARS* Advanced respiratory support, *NA* Not applicable, *SD* standard deviation

The children received a mean of 3.1 ± 1.9 medical care procedures routinely. The most frequent procedures were enteral nutrition (via gastrostomy, jejunostomy, or nasogastric tube), reported by 218 participants (78.1%), and sputum suctioning, reported by 192 participants (68.8%).

### Baseline characteristics of the ARS and non-ARS groups

Bivariate analysis showed significant differences in several clinical characteristics between the ARS group (i.e., children for whom the interruption of medical care directly affects prognosis and physical stability) and the non-ARS group. The ARS group had a significantly higher mean number of medical care procedures (3.8 ± 1.6; *p* < 0.001) compared with the non-ARS group. Furthermore, the proportions of children requiring tracheostomy management (*n* = 114, 97.4%; *p* < 0.001) and enteral nutrition (via gastrostomy, jejunostomy, or nasogastric tube) (*n* = 177, 81.2%; *p* < 0.001) were significantly higher in the ARS group (Table 1).

**Fig. 1:**
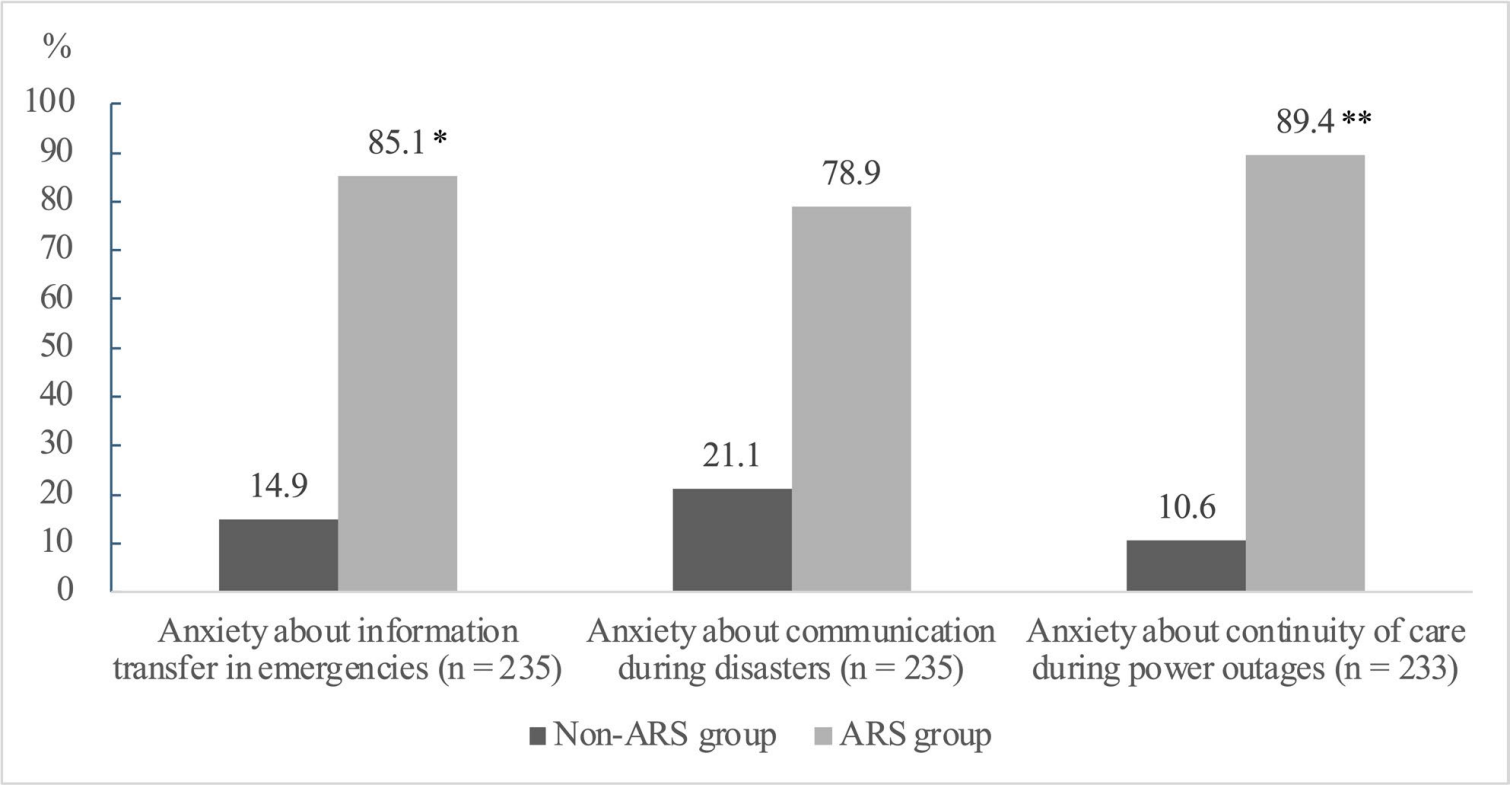
Anxiety about information transfer, communication, and care continuity during disasters/emergencies: ARS vs. non-ARS group. *p = 0.033, **p < 0.001. ARS Advanced respiratory support

Regarding disaster-related concerns, the ARS group demonstrated significantly higher levels of anxiety across multiple domains (Fig. 1). Specifically, caregivers in the ARS group were significantly more likely to report anxiety about accurately communicating their child’s condition and care requirements to unfamiliar medical professionals during emergencies (*n* = 57, 85.1%: *p* = 0.033). Additionally, anxiety about the continuity of medical care during disaster-related power outages was significantly more prevalent in the ARS group (*n* = 144, 89.4%; *p* < 0.001).

### Independent effect of ARS on disaster-related anxiety

The results of the binomial logistic regression analysis, with each type of disaster-related anxiety as a dependent variable, showed that requiring ARS had a significant independent effect on specific anxieties. Specifically, compared with the non-ARS group, the ARS group was significantly more likely to experience anxiety regarding information transfer to unfamiliar professionals (adjusted odds ratio [AOR]: 2.60, 95% confidence interval [CI]: 1.13–5.99; *p* = 0.025). Furthermore, regarding anxiety about continuity of care during power outages, the ARS group demonstrated markedly higher odds compared to the non-ARS group (AOR: 5.87, 95% CI: 2.99–11.52; *p* < 0.001) (Table 2).

**Table 2:** Independent effect of requiring ARS on the three types of disaster-related anxiety

	*n* (%) or mean ± SD	Anxiety about information transfer to unfamiliar professionals (*n* = 235)		Anxiety about loss of communication methods (*n* = 235)		Anxiety about continuity of care during power outages (*n* = 233)	
Primary independent variable		COR (95% CI)	AOR (95% CI)	COR (95% CI)	AOR (95% CI)	COR (95% CI)	AOR (95% CI)
ARS group (ref: No)	78 (33.2%)	2.20 (1.05–4.59)	2.60 (1.13–5.99) *	1.27 (0.55–2.92)	0.99 (0.42–2.36)	6.42 (3.44–12,00)	5.87 (2.99–11.52) **
Covariates							
Caregiver’s age	42.5 ± 8.2	0.99 (0.96–1.03)	0.98 (0.93–1.03)	1.00 (0.96–1.05)	0.99 (0.93–1.05)	0.10 (1.00–1.03)	0.98 (0.93–1.03)
Child’s age	9.5 ± 7.0	1.01 (0.97–1.05)	1.00 (0.94–1.07)	1.01 (0.96–1.06)	1.03 (0.96–1.12)	1.01 (0.97–1.04)	1.01 (0.95–1.07)
Evacuation method (ref: No)	166 (70.6%)	0.62 (0.34–1.12)	0.50 (0.24–1.04)	1.60 (0.67–3.81)	1.68 (0.60–4.70)	0.77 (0.44–1.34)	0.63 (0.30–1.31)
Satisfaction with information sharing with support providers (ref: Yes)	44 (18.7%)	0.48 (0.27–0.84)	0.55 (0.30–1.03)	1.10 (0.55–2.17)	0.92 (0.43–1.94)	1.13 (0.70–1.83)	1.22 (0.69–2.06)

Binary logistic regression analysis

*AOR* Adjusted odds ratio, *ARS* Advanced respiratory support, *CI* Confidence interval, *COR* Crude odds ratio, *SD* standard deviation

*p = 0.025 and **p < 0.001

### Specific concerns and preparations regarding information transfer

Regarding specific anxieties about information transfer (Q22), 60.2% of the respondents worried “Whether I might be too upset to explain things effectively,” and 33.7% expressed “Difficulty in explaining medical equipment or specialized care verbally.” Furthermore, regarding preparations for smooth information transfer (Q23), caregivers reported proactive measures such as “Carrying documents summarizing the child’s medical information” (30.3%) and “Saving medical information on a smartphone” (14.2%) (Table 1 in Additional File 2 and Table 2 in Additional File 3). These findings indicate the practical challenges caregivers face when attempting to share complex medical information with unfamiliar supporters during emergencies.

## Discussion

In this study conducted in Japan, a country with a high frequency of earthquakes and increasing climate-related disasters [35, 36], we quantitatively demonstrated for the first time that caregivers of children requiring ARS experience a significantly higher likelihood of anxiety about specific aspects of disaster situations. A key finding was that, even after adjusting for covariates, the ARS group had approximately 2.6-fold higher odds of anxiety about information transfer to unfamiliar professionals, and approximately 5.9-fold higher odds of anxiety about continuity of care during power outages compared with the non-ARS group. Conversely, no significant difference was observed regarding anxiety about loss of communication methods. The descriptive data on caregivers’ specific concerns and preparations support the existence of this information asymmetry. As noted, many caregivers fear being too upset to communicate effectively or are worried about the difficulty of verbally explaining complex medical equipment. The proactive measures they engage in, such as carrying summary medical documents or saving data on smartphones, directly reflect their daily struggle to bridge the substantial information gap between themselves and unfamiliar supporters. This underpins our interpretation that information asymmetry is a core driver of caregivers’ disaster-related anxiety. These findings highlight the severity of the information asymmetry experienced by parents in the ARS group. Specifically, the complex care needs of their child, which include highly individualized, detailed procedures and practical “know-how,” have been internalized by caregivers through their extensive experience. This creates a substantial information gap between caregivers and support providers. This asymmetry may delay appropriate requests for assistance during power outages, thereby increasing anxiety about potential life-threatening risks to the child.

Regarding the higher anxiety about information transfer to unfamiliar professionals among caregivers in the ARS group, it is possible that their dependence on medical devices such as ventilators plays an important role. Although maintaining life while performing medical procedures is part of these caregivers’ daily routines, such care is characterized by a high level of individuality [29]. Previous studies have shown that home care for CMC is complex and diverse rather than uniform, even among CMC with the same primary diagnosis or disability [37]. Specifically, care varies depending on the child’s medical condition, the devices used, family support systems, and economic status, with caregivers developing their own unique coping strategies [29, 38]. In a stressful and chaotic disaster situation, caregivers may perceive that accurately conveying these details to others is difficult, even if they do attempt to verbalize them or summarize them in writing. In particular, the existence of unique daily care routines and specific considerations known only to parents may further increase the difficulty of communication and intensify their anxiety. These findings suggest that the existence of care details and “know-how” possessed only by parents (i.e., the presence of information asymmetry) is a key factor that increases caregivers’ anxiety. The lack of a significant difference between the two groups regarding anxiety about situations where communication methods are unavailable suggests that the fear of losing basic communication infrastructure (e.g., mobile phone networks) is universally high among all caregivers of CMC, regardless of their dependence on ARS.

In this study, the risk of anxiety about care continuity during power outages was extremely high in the ARS group, approximately 5.9 times higher than in the non-ARS group. While power outages during disasters cause major inconvenience to the general public, for caregivers in the ARS group, such outages are experienced as a life-threatening crisis owing to the potential failure of life-sustaining equipment such as ventilators. This absolute dependence on securing electricity for life maintenance likely underlies this high odds ratio. Indeed, it has been repeatedly shown that power outages associated with disasters threaten life maintenance and cause substantial anxiety and stress for caregivers of CMC [39, 40, 41]. The results regarding anxiety about information transfer to unfamiliar professionals in this study indicate that this anxiety may extend beyond the mere difficulty of maintaining medical equipment in situations of power loss. To secure a power source, caregivers must explain and negotiate the need for priority access to electricity with supporters at home or in evacuation centers. Caregivers in the ARS group may fear that if they cannot accurately communicate their complex needs, they will not be granted priority power access, making it difficult to secure the necessary environment and considerations for continuation of medical care. This may create a profound fear of being unable to access the medical care needed to maintain daily life. Such anxieties about information transfer to unfamiliar professionals and the fear of being refused at evacuation centers may also affect evacuation behavior. Previous studies have reported that caregivers of CMC often hold negative views of evacuation because the complexity of care and daily life concerns make it difficult to maintain medical care at evacuation sites [42, 43]. Furthermore, caregivers of CMC may experience feelings of despair if they believe that they cannot expect support from neighbors or others during disasters and must simply give up [15], and they fear being refused acceptance at evacuation centers [44]. The anxiety about power outages among caregivers in the ARS group demonstrated in this study provides quantitative evidence for the structural vulnerability caused by information asymmetry, a situation characterized by isolation during emergencies and the reality that only caregivers themselves understand the complex care their child requires (and these care needs are difficult to explain to others).

To overcome the barriers created by the “communication wall” and “power outage anxiety” identified in this study, it is suggested that the current system, which relies on oral explanations by caregivers during dangerous disaster conditions, must be revised. Although previous studies have noted the communication difficulties experienced by both caregivers and supporters [45], the results of the present study quantitatively confirm that this challenge manifests as extremely high psychological anxiety, particularly in caregivers of CMC requiring advanced ARS. One effective solution to these challenges is the social implementation of information-sharing systems that use digital tools such as smartphones [46, 47]. It has been suggested that the use of personal health records improves access to medical information and promotes continuity of care during disasters [48]. However, proposing the use of electricity-dependent digital tools to caregivers who express substantial anxiety about power outages is inherently paradoxical. To ensure the appropriateness and safety of this approach, such digital systems must be designed with offline capabilities (e.g., local data caching) to guarantee access to vital information even when communication networks or power supplies are disrupted. Furthermore, the practical implementation of these tools must involve the securing of backup power sources, such as high-capacity mobile batteries, and they should be considered a complement to traditional paper-based analog records rather than a complete replacement. If a system were available that enabled caregivers to input necessary care information into a smartphone and manage it via the cloud during stable periods—allowing unfamiliar medical personnel or evacuation center staff to instantly access this information during emergencies—the anxiety about the inability to communicate the care needs of CMC could be greatly mitigated. In fact, a system introduced in the United States showed that the use of mHealth applications contributed to the accuracy of tracking patient movements and needs, as well as triage, in hurricane scenarios [49]. Such digital “advocacy through information” could facilitate negotiations for power access and smooth triage processes, ultimately contributing to the establishment of rapid relief and support systems to protect the lives of children who require ARS.

This study had several limitations. First, as the survey was conducted online and participants were recruited through specific advocacy groups, selection bias cannot be ruled out. The respondents likely represent a population with higher digital literacy or a pre-existing interest in information sharing; thus, the findings may not reflect the situation of caregivers who do not use digital tools or who have fewer connections with relevant organizations and local governments. Second, data for the primary outcome, anxiety, were based on subjective self-reports by caregivers. Because these results represent participants’ perceptions, they may be affected by biases related to individual personality traits or temperaments. Furthermore, because we did not measure caregivers’ baseline habitual anxiety, it is difficult to determine whether the heightened anxiety observed in the ARS group is actually disaster-specific or mainly reflects the persistent, high-stakes stress inherent in managing life-sustaining equipment. Third, although this was a nationwide survey in Japan, it did not account for the geographical vulnerability of the respondents’ residential areas to specific disasters. Furthermore, socioeconomic factors, such as household income, were not included in the analysis. Fourth, most respondents provided answers based on anticipated anxiety about potential disaster situations rather than lived experiences during actual events. Anticipated anxiety may differ from the actual anxiety experienced during a large-scale disaster. Further confirmation is needed to determine the actual effectiveness of information sharing during a real disaster event. Fifth, the survey instrument was developed based on literature reviews and discussions among collaborators. Structured consensus and validation procedures, such as the Delphi method, were not used. Therefore, potential researcher expectancy effects and limitations regarding content validity and reproducibility cannot be entirely ruled out. Finally, it is important to note that the unadjusted group comparisons presented in Table 1; Fig. 1 may be substantially affected by confounding factors such as the child’s age and evacuation difficulty. Therefore, the independent effect of requiring ARS on disaster-related anxiety should be interpreted solely based on the AORs from the binomial logistic regression analysis (Table 2), and caution is required when interpreting the bivariate analysis results. This study used a cross-sectional design; although an association between ARS care needs and anxiety was identified, this does not prove a cause-and-effect relationship. Therefore, caution is required in generalizing these results.

## Conclusion

This study quantitatively demonstrated that caregivers of CMC requiring ARS experience a significantly higher risk of some types of anxiety compared with caregivers of CMC who do not require ARS. In the ARS group, anxiety about the accurate transmission of information to medical professionals met for the first time was approximately 2.6 times higher, and anxiety about continuity of care during power outages was 5.9 times higher. However, anxiety about the transmission of information when communication methods are unavailable showed no significant difference, indicating that this was a shared, fundamental concern among all caregivers. The results suggest that information asymmetry (i.e., only caregivers possess detailed knowledge of the child’s care) creates perceived difficulties in communication during disasters and intensifies the fear of being unable to maintain life-sustaining medical care during power outages. To address these challenges, there is a clear need for the social implementation of personal health records for CMC, using digital devices such as smartphones to complement traditional oral and paper-based methods. The routine sharing and accumulation of information during stable periods could mitigate caregivers’ anxiety and contribute to more rapid and effective responses during emergencies such as disasters.

## Supplementary Information

Additional file 1: Survey Questionnaire.

Additional file 2: Table 1. Specific concerns regarding information transfer (Q22).

Additional file 3: Table 2. Preparations for smooth information transfer (Q23).

## Data Availability

The datasets generated and analyzed during the current study are not publicly available owing to the sensitive nature of the information obtained from caregivers of children with medical complexity and to ensure the privacy of the participants. However, they are available from the corresponding author on reasonable request.

## Abbreviations

CMC: Children with Medical Complexity
ARS: Advanced Respiratory Support
AOR: Adjusted Odds Ratio
CI: Confidence Interval

## References

[1] Berry JG, Hall M, Cohen E, O’Neill M, Feudtner C. Ways to Identify Children with Medical Complexity and the Importance of Why. J Pediatr. 2015;167:229–37. 10.1016/j.jpeds.2015.04.068. 26028285 10.1016/j.jpeds.2015.04.068 PMC5164919

[2] Gallo M, Agostiniani R, Pintus R, Fanos V. The child with medical complexity. Ital J Pediatr. 2021;47:1. 10.1186/s13052-020-00935-z. 33407754 10.1186/s13052-020-00935-z PMC7788740

[3] Abraham G, Fehr J, Ahmad F, Jeffe DB, Copper T, Yu F, et al. Emergency Information Forms for Children With Medical Complexity: A Simulation Study. Pediatrics. 2016;138:e20160847. 10.1542/peds.2016-0847. 27436504 10.1542/peds.2016-0847 PMC5603153

[4] Kuo DZ, McAllister JW, Rossignol L, Turchi RM, Stille CJ. Care Coordination for Children With Medical Complexity: Whose Care Is It. Anyway? Pediatr. 2018;141:S224–32. 10.1542/peds.2017-1284G. 10.1542/peds.2017-1284G 29496973

[5] Yamaoka Y, Tamiya N, Watanabe A, Miyazono Y, Tanaka R, Matsuzawa A, et al. Hospital-based care utilization of children with medical complexity in Japan. Pediatr Int. 2018;60:626–33. 10.1111/ped.13586. 29676518 10.1111/ped.13586

[6] Allshouse C, Comeau M, Rodgers R, Wells N. Families of Children With Medical Complexity: A View From the Front Lines. Pediatrics. 2018;141:S195–201. 10.1542/peds.2017-1284D. 29496970 10.1542/peds.2017-1284D

[7] Altman L, Zurynski Y, Breen C, Hoffmann T, Woolfenden S. A qualitative study of health care providers’ perceptions and experiences of working together to care for children with medical complexity (CMC). BMC Health Serv Res. 2018;18:70. 10.1186/s12913-018-2857-8. 29386026 10.1186/s12913-018-2857-8 PMC5793356

[8] Kemp L, Xu C, Depledge J, Ebi KL, Gibbins G, Kohler TA et al. Climate Endgame: Exploring catastrophic climate change scenarios. Proceedings of the National Academy of Sciences. 2022;119:e2108146119. 10.1073/pnas.2108146119. 10.1073/pnas.2108146119 PMC9407216 35914185

[9] Kawase H, Murata A, Mizuta R, Sasaki H, Nosaka M, Ishii M, et al. Enhancement of heavy daily snowfall in central Japan due to global warming as projected by large ensemble of regional climate simulations. Clim Change. 2016;139:265–78. 10.1007/s10584-016-1781-3.

[10] Shimizu K, Asahiro K. The influence of orchard and forest management on rainfall-induced landslides: A study of Hiraenoki Community in southwestern Japan. Trees Forests People. 2026;23:101103. 10.1016/j.tfp.2025.101103.

[11] Newman M, Leochico CFD. Promoting disaster preparedness for children with special healthcare needs: A scoping review. J Clim Change Health. 2022;8:100145. 10.1016/j.joclim.2022.100145.

[12] Matsushita S, Tanaka T, Sogo Y, Manami S, Nakagawa T, Tanida K, et al. Caregivers’ perspectives on disaster preparedness and evacuation plan for children relying on home ventilators or home oxygen. Int J Disaster Risk Reduct. 2024;108:104534. 10.1016/j.ijdrr.2024.104534.

[13] Mourid MR, Abdelrahman ST, Aziz MM, Eldosoky AA, Mousa MM, Ebraheem EA, et al. Why Pediatric Emergency Departments Are not Ready for Disasters. Curr Emerg Hosp Med Rep. 2025;13:16. 10.1007/s40138-025-00320-2.

[14] Nakayama T, Tanaka S, Uematsu M, Kikuchi A, Hino-Fukuyo N, Morimoto T, et al. Effect of a blackout in pediatric patients with home medical devices during the 2011 eastern Japan earthquake. Brain Develop. 2014;36:143–7. 10.1016/j.braindev.2013.02.001. 10.1016/j.braindev.2013.02.001 23452913

[15] Smith N, Donaldson M, Mitton C, Lee E. Communication in disasters to support families with children with medical complexity and special healthcare needs: a rapid scoping review. Front Public Health. 2024;12:1229738. 10.3389/fpubh.2024.1229738. 38544735 10.3389/fpubh.2024.1229738 PMC10967951

[16] Hipper TJ, Davis R, Massey PM, Turchi RM, Lubell KM, Pechta LE, et al. The Disaster Information Needs of Families of Children with Special Healthcare Needs: A Scoping Review. Health Secur. 2018;16:178–92. 10.1089/hs.2018.0007. 29883200 10.1089/hs.2018.0007 PMC11015856

[17] Ciurria JA, Lin JR, Pruitt CM, Sisk BA. Functions of communication during emergency care of children with medical complexity: Caregiver perspectives. Patient Educ Couns. 2025;134:108667. 10.1016/j.pec.2025.108667. 39842067 10.1016/j.pec.2025.108667

[18] Nonoyama T, Kadoma A, Kaneko T, Niinomi K, Ozaki I, Asano M. Psychosocial support and care for children with special healthcare needs and their families: A scoping review for enhancing the care system. Medicine. 2025;104:e41944. 10.1097/MD.0000000000041944. 40128054 10.1097/MD.0000000000041944 PMC11936650

[19] Baumbusch J, Fong V, Lee E, Bandara NA, Khan KB. Weathering the Storm: Climate-Related Weather Event Experiences of Families of Children With Medical Complexity. Child Care Health Dev. 2025;51:e70068. 10.1111/cch.70068. 40102035 10.1111/cch.70068 PMC11919613

[20] Ogihara H, Watanabe Y. Are disaster support measures for children with medical complexities in depopulated areas adequate? A pediatric nursing perspective based on a local government survey. J Pediatr Nurs. 2025;85:607–15. 10.1016/j.pedn.2025.09.023. 41045762 10.1016/j.pedn.2025.09.023

[21] Prasser A, Beckert J, Köhler M, Ewers M. Complex Care Needs of People with Technology Dependence in Disaster Situations: A Scoping Review. Healthcare. 2025;13:3305. 10.3390/healthcare13243305. 41464373 10.3390/healthcare13243305 PMC12732631

[22] Ghandour RM, Hirai AH, Kenney MK. Children and Youth With Special Health Care Needs: A Profile. Pediatrics. 2022;149(Supplement 7):e2021056150D. 10.1542/peds.2021-056150D. 35642877 10.1542/peds.2021-056150D

[23] Dewan T, Birnie K, Drury J, Jordan I, Miller M, Neville A, et al. Experiences of medical traumatic stress in parents of children with medical complexity. Child Care Health Dev. 2023;49:292–303. 10.1111/cch.13042. 35947493 10.1111/cch.13042 PMC10087969

[24] Nakai H, Tsukasaki K, Kyota K, Itatani T, Nihonyanagi R, Shinmei Y, et al. Factors Related to Evacuation Intentions of Power-Dependent Home Care Patients in Japan. J Commun Health Nurs. 2016;33:196–208. 10.1080/07370016.2016.1227213. 10.1080/07370016.2016.1227213 27749088

[25] Matsumoto Y, Nakai H, Koga Y, Hasegawa T, Miyagi Y. Disaster Evacuation for Home-Based Patients with Special Healthcare Needs: A Cross-Sectional Study. Int J Environ Res Public Health. 2022;19:15356. 10.3390/ijerph192215356. 36430076 10.3390/ijerph192215356 PMC9690564

[26] Cabinet Office. Revised Guideline for Supporting Evacuation of Persons Requiring Assistance in Evacuation (in Japanese). 2025. https://www.bousai.go.jp/taisaku/hisaisyagyousei/youengosya/r3/pdf/shishin_r7.pdf. Accessed 9 Apr 2026.

[27] Children and Families Agency. Support measures for children with medical complexity and others, and their families(in Japanese). 2024. https://www.cfa.go.jp/policies/shougaijishien/care-ji-shien. Accessed 18 Aug 2024.

[28] Ministry of Health, Labour and Welfare. Challenges of Information Sharing in Daily Life for Children with Medical Complexity (in Japanese). 2020. https://www.mhlw.go.jp/content/12200000/000653544.pdf. Accessed 16 Aug 2024.

[29] Pitch N, Shahil A, Mekhuri S, Ambreen M, Chu S, Keilty K, et al. Caring for children with new medical technology at home: parental perspectives. bmjpo. 2023;7:e002062. 10.1136/bmjpo-2023-002062. 10.1136/bmjpo-2023-002062 PMC10603509 37865398

[30] Quigley L, Lacombe-Duncan A, Adams S, Moore Hepburn C, Cohen E. A qualitative analysis of information sharing for children with medical complexity within and across health care organizations. BMC Health Serv Res. 2014;14:283. 10.1186/1472-6963-14-283. 24981205 10.1186/1472-6963-14-283 PMC4085394

[31] Harvey AR, Meehan E, Merrick N, D’Aprano AL, Cox GR, Williams K, et al. Comprehensive care programmes for children with medical complexity. Cochrane Database Syst Rev. 2024;2024:CD013329. 10.1002/14651858.CD013329.pub2. 10.1002/14651858.CD013329.pub2 PMC11137836 38813833

[32] Yun H-J, Parker ML, Wilson CB, Cui M. Medical Complexity of Children with Special Healthcare Needs and Healthcare Experiences. Child (Basel). 2024;11:775. 10.3390/children11070775. 10.3390/children11070775 PMC11275203 39062228

[33] DellaBadia K, Tauber D. Respiratory concerns in children with medical complexity. Curr Probl Pediatr Adolesc Health Care. 2021;51:101072. 10.1016/j.cppeds.2021.101072. 34657813 10.1016/j.cppeds.2021.101072

[34] Batson L, Donohue PK, Boss RD, Seltzer RR. Family challenges in personal transportation of children with medical complexity. J Pediatr Rehabil Med. 2022;15:655–65. 10.3233/PRM-220015. 36502348 10.3233/PRM-220015

[35] Hayashi S, Kunitomo M, Mikami K, Suzuki K. Recent and Historical Background and Current Challenges for Sediment Disaster Measures against Climate Change in Japan. Water. 2022;14:2285. 10.3390/w14152285.

[36] Japan Meteorological Agency. Earthquake information. 2026. https://www.data.jma.go.jp/multi/quake/index.html?lang=en. Accessed 6 Feb 2026.

[37] Maeda H, Tomomatsu I, Iikura I, Ikari M, Kondo Y, Yamamoto M, et al. The care burden for technology-dependent children with long-term home ventilation increases along with the improvement of their motor functions. Eur J Pediatr. 2024;183:135–47. 10.1007/s00431-023-05249-w. 37843613 10.1007/s00431-023-05249-w PMC10858118

[38] Toly VB, Blanchette JE, Al-Shammari T, Musil CM. Caring for technology-dependent children at home: Problems and solutions identified by mothers. Appl Nurs Res. 2019;50:151195. 10.1016/j.apnr.2019.151195. 31668894 10.1016/j.apnr.2019.151195 PMC7857638

[39] Lehmann PS. Healthcare System Responses to Blackouts and Natural Disasters. 2024. https://ihsonline.org/Portals/0/Tech%20Papers/2024_Papers/Lehmann_Healthcare_System_Responses_to_Blackouts_and_Natural_Disasters.pdf?ver=Sl07S_1vN-pFARVRjMIILw%3D%3D. Accessed 7 Feb 2026.

[40] Spurlock T, Sewell K, Sugg MM, Runkle JD, Mercado R, Tyson JS, et al. A spatial analysis of power-dependent medical equipment and extreme weather risk in the southeastern United States. Int J Disaster Risk Reduct. 2023;95:103844. 10.1016/j.ijdrr.2023.103844. 39309639 10.1016/j.ijdrr.2023.103844 PMC11414598

[41] Casey JA, Fukurai M, Hernández D, Balsari S, Kiang MV. Power outages and community health: a narrative review. Curr Environ Health Rep. 2020;7:371–83. 10.1007/s40572-020-00295-0. 33179170 10.1007/s40572-020-00295-0 PMC7749027

[42] Matsumoto Y, Nakai H, Koga Y, Hasegawa T, Miyagi Y. Disaster Evacuation for Home-Based Patients with Special Healthcare Needs: A Cross-Sectional Study. Int J Environ Res Public Health. 2022;19:15356. 10.3390/ijerph192215356. 36430076 10.3390/ijerph192215356 PMC9690564

[43] Nakai H, Tsukasaki K, Kyota K, Itatani T, Nihonyanagi R, Shinmei Y, et al. Factors Related to Evacuation Intentions of Power-Dependent Home Care Patients in Japan. null. 2016;33:196–208. 10.1080/07370016.2016.1227213. 10.1080/07370016.2016.1227213 27749088

[44] Seltzer RR, Thompson BS, Feudtner C. The Daunting Problem of Medical Complexity and Housing Instability. Pediatrics. 2020;146:e20193284. 10.1542/peds.2019-3284. 32561611 10.1542/peds.2019-3284

[45] Nakai H, Itatani T, Horiike R. Application Software That Can Prepare for Disasters Based on Patient-Participatory Evidence: K-DiPS: A Verification Report. Int J Environ Res Public Health. 2022;19:9694. 10.3390/ijerph19159694. 35955050 10.3390/ijerph19159694 PMC9368305

[46] Stampe K, Kishik S, Müller SD. Mobile Health in Chronic Disease Management and Patient Empowerment: Exploratory Qualitative Investigation Into Patient-Physician Consultations. J Med Internet Res. 2021;23:e26991. 10.2196/26991. 34128817 10.2196/26991 PMC8277350

[47] Sousa P, Martinho R, Parreira P, Luo G. Editorial: mHealth tools for patient empowerment and chronic disease management. Front Psychol. 2023;14:1206567. 10.3389/fpsyg.2023.1206567. 37388655 10.3389/fpsyg.2023.1206567 PMC10305777

[48] Paydar S, Emami H, Asadi F, Moghaddasi H, Hosseini A. Functions and Outcomes of Personal Health Records for Patients with Chronic Diseases: A Systematic Review. Perspect Health Inf Manag. 2021;18(Spring):1l. 34345228 PMC8314040

[49] Abualenain J. Use of Technology in Disaster Medicine. Eurasian J Emerg Med. 2024;23:155–8. 10.4274/eajem.galenos.2024.45452.

