# Supplementary material for: Effect of requiring advanced respiratory support on disaster-related anxiety among caregivers of children with medical complexity: a cross-sectional study
Source: BMC Public Health. 2026 May 7;26:1978. doi: 10.1186/s12889-026-27601-z (PMC13321626; doi:10.1186/s12889-026-27601-z)
Supplement: Supplementary file 1 — Additional file 1: Survey Questionnaire. [file 12889_2026_27601_MOESM1_ESM.docx]

**Additional File 1: Survey Questionnaire**

**Title:** Questionnaire Survey on Information-Sharing Challenges among Caregivers of Children with Medical Complexity (Online Survey)

1. About the Respondent and Your Child

- Please select the prefecture where you currently reside
- [Dropdown list of all 47 Japanese prefectures]

1. Please select the gender identity that best describes you
   - Male
   - Female
   - Other
   - Prefer not to say
2. Please select your age group at the time of this survey
   - 10s (19 years old or younger)
   - 20s (20–29 years old)
   - 30s (30–39 years old)
   - 40s (40–49 years old)
   - 50s (50–59 years old)
   - 60s (60–69 years old)
   - 70s or older (70 years old or older)
3. For more detailed statistical analysis (e.g., calculating the mean age), please enter your current age in years. This question is optional
4. What is your relationship to the child?
   - Mother
   - Father
   - Other (Please specify: )
5. Please select your child’s gender
   - Male
   - Female
   - Other
   - Prefer not to say
6. Please enter your child’s current age. If the child is under 1 year old, please also provide the age in months
7. Please select all types of medical care your child routinely receives (Select all that apply)
   - Being carried by a caregiver
   - Manual wheelchair
   - Electric wheelchair
   - Adaptive stroller
   - Walker
   - Ambulation without assistance
   - Other (Please specify: )
8. What method(s) do you plan to use for your child’s movement during an evacuation? (Select all that apply)
   - Being carried by a caregiver
   - Manual wheelchair
   - Electric wheelchair
   - Adaptive stroller
   - Walker
   - Ambulation without assistance
   - Other (Please specify: )
9. Please select all types of medical care your child routinely receives (Select all that apply)
   - Mechanical ventilation (via tracheostomy)
   - Non-invasive ventilation (e.g., via oral or nasal mask)
   - High-flow nasal cannula oxygen therapy
   - Mechanical insufflation-exsufflation (e.g., Cough Assist, intrapulmonary percussive ventilation)
   - Tracheostomy management
   - Suctioning (oral, nasal, or tracheal)
   - Enteral nutrition (via gastrostomy, jejunostomy, or nasogastric tube)
   - Total parenteral nutrition (via central venous catheter)
   - Management of urinary catheterization (clean intermittent or continuous)
   - Stoma management (e.g., colostomy or ileostomy)
   - Home oxygen therapy
   - Insulin injections
   - Routine blood glucose monitoring
   - Other self-injections (Please specify: )
   - Peritoneal dialysis
   - None of the above
   - Other (Please specify: )
10. Please describe your child’s primary diagnosis or medical condition (Optional)
11. Have you ever experienced evacuating with your child during a disaster?
    - No
    - Yes, evacuated to a shelter or similar location
    - Yes, performed vertical evacuation within my home
    - Other (Please specify: )

**Daily Information Sharing with Support Providers**

1. Please select all support providers or organizations with whom you routinely share information regarding your child’s care and daily life (Select all that apply)
   - Attending physician at a clinic (e.g., pediatrician)
   - Home-visit physician
   - Hospital physician (e.g., at a university hospital)
   - Nurses from a home-visit nursing station
   - Home-visit pharmacist
   - Hospital nurses
   - Medical social worker
   - Dentist / Dental hygienist
   - Rehabilitation professionals (PT, OT, ST)
   - Public health nurse
   - Consultation support specialist
   - Staff at service providers (e.g., child development support, after-school day service, life care, short-stay)
   - Staff at nursery schools, kindergartens, or certified centers for early childhood education and care
   - Teachers at elementary, junior high, or special needs schools
   - Nurses at elementary, junior high, or special needs schools
   - Home-help service staff (helpers)
   - Staff at support centers for children with medical complexity
   - Medical equipment or sanitary supply providers
   - None in particular
   - Other (Please specify: )
2. What methods do you primarily use for sharing information with the support providers mentioned above? (Select all that apply)
   - Face-to-face conversation
   - Telephone
   - Communication notebooks or logbooks
   - Email
   - SNS applications (e.g., LINE)
   - Dedicated information-sharing applications/systems
   - Documents such as medical information forms (referral letters)
   - Other (Please specify: )
3. How satisfied are you with the overall daily information sharing with support providers?
   - Very dissatisfied
   - Somewhat dissatisfied
   - Neither satisfied nor dissatisfied
   - Somewhat satisfied
   - Very satisfied
4. Do you feel there are challenges regarding daily information sharing with support providers?
   - Not at all
   - Not much
   - Neither agree nor disagree
   - Somewhat
   - Significantly
5. (For those who answered “Somewhat” or “Significantly” to question 16) What specific challenges do you feel? (Select all that apply)
   - Having to explain the same information repeatedly to multiple providers
   - Insufficient information sharing among providers
   - Lack of coordination among providers
   - Communication and coordination take too much time
   - Limited means of communication (e.g., only by phone, only available during daytime)
   - Difficult to understand due to excessive medical jargon
   - Difficult to convey my intentions accurately
   - Burden of writing in records (e.g., communication notebooks)
   - Having to write the same information multiple times in records
   - The information-sharing tools being used are difficult to use
   - Having multiple information-sharing tools is cumbersome
   - Other (Please specify: )
6. Please describe any points regarding daily information sharing that you find helpful or successful (Open-ended)

**Information Transfer regarding Changes in Your Child’s Medical Condition**

1. Do you have an emergency contact list or similar resources prepared in case your child’s condition suddenly worsens or signs of deterioration appear?
   - No
   - Yes
2. Who do you usually contact first if your child’s medical condition worsens? (Select the most applicable one)
   - Attending physician
   - Home-visit nurse
   - Emergency services (119)
   - Family members / Relatives
   - Other (Please specify: )
3. Are you anxious about accurately communicating your child’s condition and care requirements to medical professionals meeting your child for the first time (e.g., emergency medical technicians, emergency department physicians, or nurses) during an emergency, such as during an ambulance transport?
   - Not at all
   - Not much
   - Somewhat
   - Significantly
4. (For those who answered “Somewhat” or “Significantly” to question 21) What specific points are you anxious about? (Select all that apply)
   - Whether I can convey all necessary information without omission within a limited time
   - Whether I might be too upset to explain things effectively
   - Difficulty in organizing the information to be conveyed (e.g., medical history, allergies, care details, medications)
   - Whether the responder will understand the situation if they lack specialized knowledge about children
   - Difficulty in explaining medical equipment or specialized care verbally
   - Whether they will understand the care needed in an unusual situation (e.g., frequency or method of suctioning)
   - Other (Please specify: )
5. What resources have you prepared to facilitate smooth information transfer when your child’s condition worsens? (Select all that apply)
   - Creating an emergency contact list
   - Carrying documents summarizing the child’s medical information (diagnosis, allergies, current medications, etc.)
   - Saving medical information on a smartphone
   - Saving medical information on a tablet device
   - Utilizing “Help Cards/Marks” or related goods
   - Keeping daily health and care records in a notebook for immediate access
   - Keeping daily health and care records on a smartphone for immediate access
   - Participating in drills and simulations (e.g., who to contact and what to convey)
   - None in particular
   - Other (Please specify: )

**Information Transfer during Disasters (Including Imminent Disaster Threats)**

1. How do you prepare and manage information regarding your child’s care for disasters such as earthquakes, typhoons, heavy rain, infectious diseases, or power outages? (Select all that apply)
   - Preparing documents/copies summarizing medical information (similar to those for emergencies)
   - Preparing notes summarizing care procedures
   - Preparing an emergency contact list
   - Creating a stockpile list for medications, medical supplies, and nutritional supplements
   - Stockpiling physical medications, medical supplies, and nutritional supplements
   - Preparing spare batteries for medical equipment
   - Preparing alternative power sources (e.g., storage batteries, charging from a vehicle)
   - Confirming evacuation sites and routes
   - Utilizing Individual Evacuation Plans
   - Saving information on a smartphone to ensure offline access
   - None in particular
   - Other (Please specify: )
2. Are you anxious about reporting your child’s safety or requesting rescue (to family members, supporters, or local government) during a disaster when communication methods (telephone, internet) are unavailable?
   - Not at all
   - Not much
   - Somewhat
   - Significantly
3. Are you anxious about being able to continue to receive medical care during a power outage caused by a disaster?
   - Not at all
   - Not much
   - Somewhat
   - Significantly
4. If you had to stay in an unusual location, such as an evacuation shelter, do you think you would experience difficulty in communicating information necessary for your child’s care (e.g., use of medical equipment, securing power, necessary considerations) to those around you (shelter operators, other evacuees, volunteers, etc.)?
   - Not at all
   - Not much
   - Somewhat
   - Significantly
   - Don’t know
5. What do you anticipate would be “difficulties” or “burdens” in communicating information about your child’s care during a disaster (including imminent disaster threats)? (Select all that apply)
   - Unable to communicate because communication methods are unavailable
   - Information prepared (documents, notes, etc.) is not at hand
   - Unable to secure a power source for medical equipment at the evacuation site
   - Unable to obtain cooperation for necessary care from people around
   - Lack of understanding regarding the need for private space for medical treatment at the evacuation site
   - Lack of consideration for privacy at the evacuation site
   - Being too confused to communicate information calmly
   - Information on necessary daily life support does not reach me
   - Information on public relief supplies does not reach me
   - Having to explain the child’s condition from scratch to supporters with no prior relationship
   - None in particular
   - Other (Please specify: )

**Concerns regarding Disaster Situations**

For the following items (Q29–Q33), please indicate your level of agreement or concern using the following 4-point scale:

- - Not at all
  - Not much
  - Somewhat
  - Significantly

1. Do you feel that the possibility of being unable to secure the necessary power for medical equipment during a disaster is a serious problem?
2. Do you think there is a possibility that you will not be able to obtain understanding from people around you regarding medical care (e.g., noisy equipment, frequent suctioning, required space) at an evacuation site?
3. Do you think that an Individual Evacuation Plan (created in advance with local governments and supporters according to your child’s situation) would be useful for sharing information and receiving support during a disaster?
4. If there were a dedicated information-sharing system (e.g., an application) that ensured medical information, safety confirmation, and requests for support could be reliably communicated even during a disaster, would you want to use it?
5. In the event of a disaster, if you had to verbally explain your child’s complex medical care and necessary considerations to supporters with whom you have no prior relationship, do you think you would be able to communicate calmly and accurately?

**Addressing Challenges in Information Sharing and Transfer**

1. To facilitate smoother information sharing and transfer (including in daily life, periods of worsening medical conditions, and disasters) for raising children with medical complexity, what kind of support, tools, information, or social systems do you believe should be implemented? (Open-ended)
2. If you have any other thoughts or comments you would particularly like to share in relation to this survey or your daily experiences, please feel free to write them here (Open-ended)

**Abbreviations:** **PT**, physical therapist; **OT**, occupational therapist; **ST**, speech-language-hearing therapist.
