# Supplementary material for: Effect of requiring advanced respiratory support on disaster-related anxiety among caregivers of children with medical complexity: a cross-sectional study
Source: BMC Public Health. 2026 May 7;26:1978. doi: 10.1186/s12889-026-27601-z (PMC13321626; doi:10.1186/s12889-026-27601-z)
Supplement: Supplementary file 2 — Additional file 2: Table 1. Specific concerns regarding information transfer (Q22). [file 12889_2026_27601_MOESM2_ESM.docx]

**Additional File 2**

**Table 1. Specific concerns regarding information transfer (Q22)** (Multiple answers allowed, *n* =181)

| Specific anxieties about information transfer | *n* | % |
| --- | --- | --- |
| Whether I can convey all necessary information without omission within a limited time | 147 | 81.2 |
| Whether I might be too upset to explain things effectively | 109 | 60.2 |
| Difficulty in organizing the information to be conveyed (e.g., medical history, allergies, care details, medications) | 68 | 37.6 |
| Whether the responder will understand the situation if they lack specialized knowledge about children | 96 | 53.0 |
| Difficulty in explaining medical equipment or specialized care verbally | 61 | 33.7 |
| Whether they will understand the care needed in an unusual situation (e.g., frequency or method of suctioning) | 60 | 33.1 |
| Other | 21 | 11.6 |
