# Supplementary material for: Effect of requiring advanced respiratory support on disaster-related anxiety among caregivers of children with medical complexity: a cross-sectional study
Source: BMC Public Health. 2026 May 7;26:1978. doi: 10.1186/s12889-026-27601-z (PMC13321626; doi:10.1186/s12889-026-27601-z)
Supplement: Supplementary file 3 — Additional file 3: Table 2. Preparations for smooth information transfer (Q23). [file 12889_2026_27601_MOESM3_ESM.docx]

**Additional File 3**

**Table 2. Preparations for smooth information transfer (Q23)** (Multiple answers allowed, *n* = 274)

| Preparations for smooth information transfer | n | % |
| --- | --- | --- |
| Creating an emergency contact list | 71 | 25.9 |
| Carrying documents summarizing the child’s medical information (diagnosis, allergies, current medications, etc.) | 83 | 30.3 |
| Saving medical information on a smartphone | 39 | 14.2 |
| Saving medical information on a tablet device | 8 | 2.9 |
| Utilizing “Help Cards/Marks” or related goods | 34 | 12.4 |
| Keeping daily health and care records in a notebook for immediate access | 48 | 17.5 |
| Keeping daily health and care records on a smartphone for immediate access | 20 | 7.3 |
| Participating in drills and simulations (e.g., who to contact and what to convey) | 8 | 2.9 |
| None in particular | 91 | 33.2 |
| Other | 20 | 7.3 |
